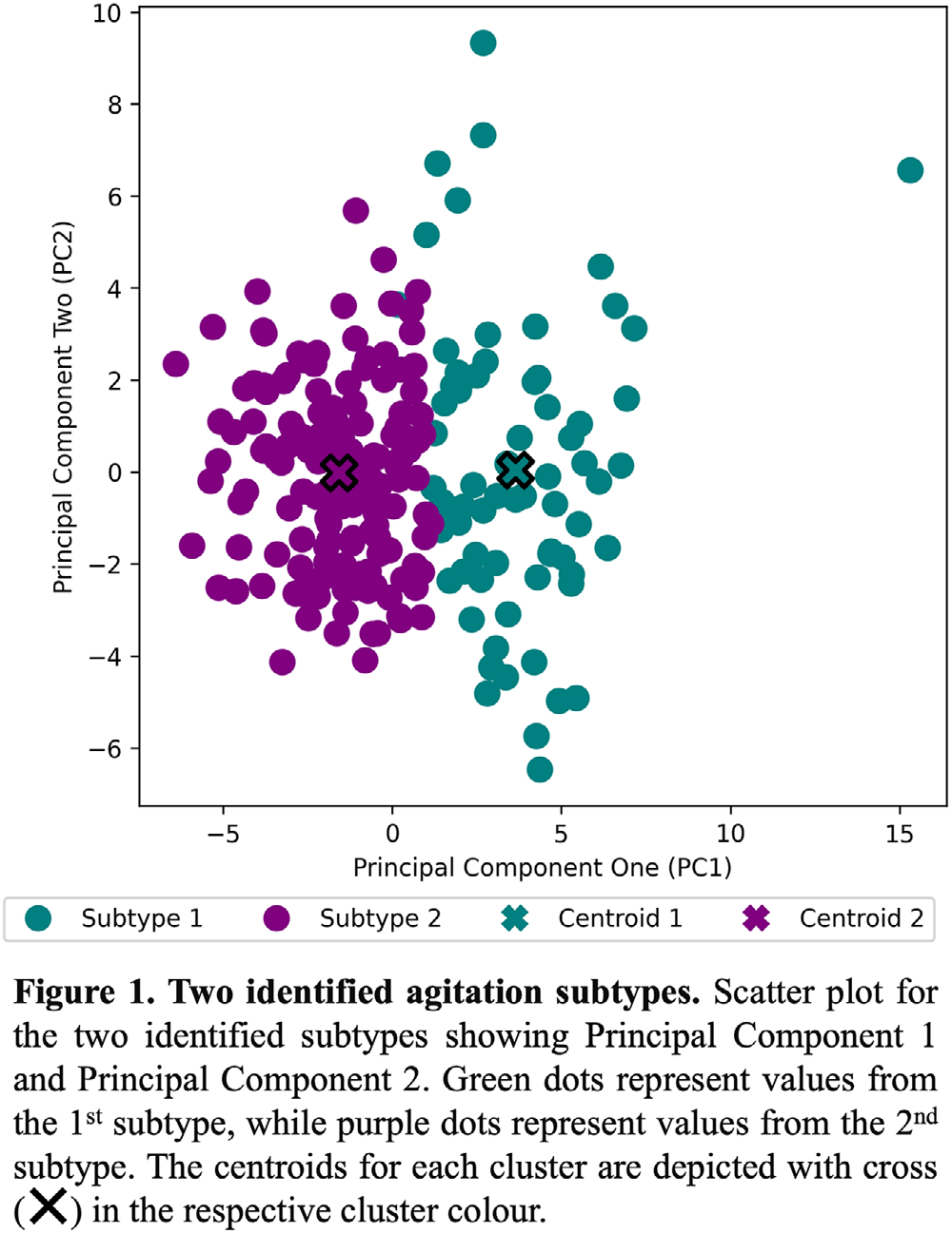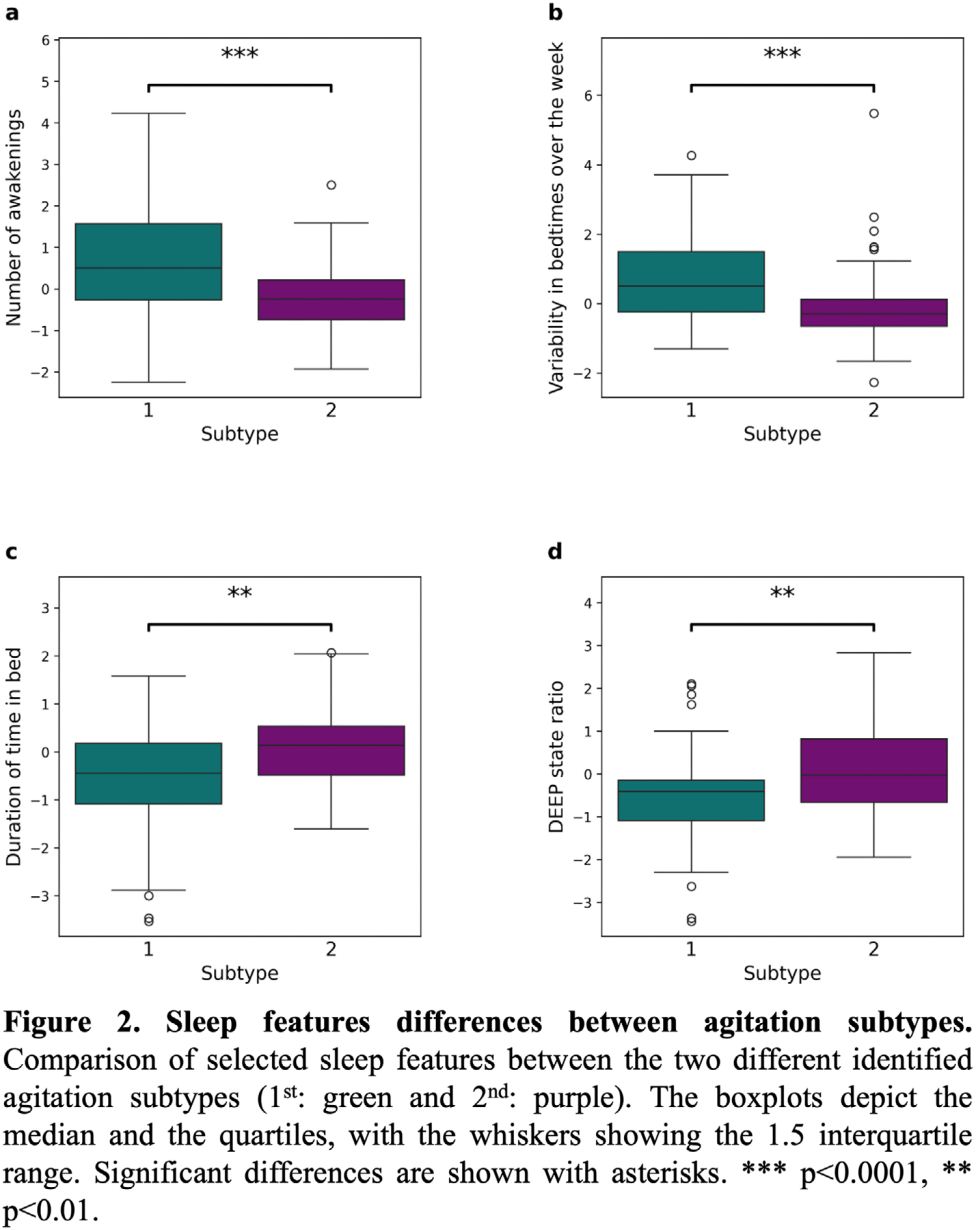# Identifying diverse agitation profiles in dementia: Insights from longitudinal in‐home monitoring data

**DOI:** 10.1002/alz.095287

**Published:** 2025-01-09

**Authors:** Marirena Bafaloukou, Ann‐Kathrin Schalkamp, Cynthia Sandor, Ramin Nilforooshan, Payam Barnaghi, CR&T Group

**Affiliations:** ^1^ Imperial College London, London United Kingdom; ^2^ UK Dementia Research Institute, Care Research and Technology Centre, London United Kingdom; ^3^ UK Dementia Research Institute, Imperial College London, London United Kingdom; ^4^ Surrey and Borders Partnership NHS Foundation Trust, Chertsey United Kingdom; ^5^ University of Surrey, Guildford United Kingdom; ^6^ Great Ormond Street Hospital NHS Foundation Trust, London United Kingdom

## Abstract

**Background:**

Agitation, affecting 30% of people living with dementia (PLwD), presents with heterogeneity, diverse triggers, and variable responses to interventions. Subtype identification particularly in real‐world settings, thus, holds promise for more tailored care.

**Method:**

We used longitudinal, in‐home monitoring data from 61 PLwD from the Minder study. 235 agitation episodes were reported, each describing the presence of agitation during an 8‐day window. Using sensor data, we derived 67 behavioural features related to activity, sleep, and physiology, normalised per PLwD to accommodate individual differences. Principal component analysis identified 14 components via Horn’s parallel analysis. We compared k‐means, Birch, Gaussian Mixture, and k‐Medoids clustering. Number of clusters was determined by Silhouette and Calinski Harabasz Score (chi). Two‐sided Welch’s t‐tests with Bonferroni correction (p*<*0.05) were used for feature comparison between subtypes. A logistic regression model was trained to predict subtypes based on 47 ambient parameters, with performance compared to a baseline model only incorporating age and sex.

**Result:**

K‐means clustering achieved the highest scores with 2 identified subtypes (chi = 34.75, silhouette = 0.21) **(Figure 1)**. 24 (63.16%) of the investigated sleep features were significantly different between the subtypes. No activity or physiological feature was significantly different. The first subtype (71 agitation episodes) was defined by more nocturnal awakenings *(p−value =* 2.60×10^−5^, *t* = 5.48*)*, increased time spent out of bed (*p*−*value* = 5.85×10^−11^ *, t* = 8*.34*) and greater variability in bedtimes *(p−value =* 7.76×10^−6^ *, t =* 5.72*)*. The second subtype (164 episodes) exhibited longer sleep durations *(p‐value =* 1.58×10^−3^, *t = 4.44)* and a higher ratio of deep sleep *(p−value =* 2.41×10^−3^, t = 4.28) **(Figure 2)**. Ambient features could predict agitation subtype (*F1‐score* = 70.98%) better than baseline *(p−value =* 2.71×10^−4^ *, t =* 4.51*)* with lounge morning illuminance contributing most. Agitation subtypes were not stable within participants with 21 (34.43%) appearing in both clusters. The subtypes did not show differences in demographics.

**Conclusion:**

Our analysis revealed two distinct behavioural agitation patterns, one associated with poor and one with good sleep, that can be predicted with ambient features. Tailoring agitation care to accommodate these subtypes could render treatment strategies more targeted and effective.